# Predicting intolerance of uncertainty in individuals with eating disorder symptoms

**DOI:** 10.1186/s40337-017-0152-4

**Published:** 2017-09-01

**Authors:** Lot C. Sternheim, Martin Fisher, Amy Harrison, Rosamond Watling

**Affiliations:** 1 Department of Psychology, Utrecht University, Utrecht, The Netherlands; 2 School of Psychotherapy & Psychology, Regent’s University London, London, UK; 3 Ellern Mede Service for Eating Disorders, London, UK; 4 Social, Health and Organisation Psychology, Faculty of Social Sciences, Utrecht University, Heidelberglaan 1, 3584 CS Utrecht, The Netherlands

**Keywords:** Intolerance of uncertainty, Eating disorders, Insecure attachment, Openness to experience, Extraversion

## Abstract

**Background:**

Intolerance of Uncertainty (IU) is recognized for its contribution to various psychopathologies, in particular anxiety and depression. Studies highlight the relevance of IU for Eating Disorders (EDs) however, potential factors contributing to IU in EDs remain unstudied.

**Methods:**

Three hundred and forty-nine women with ED symptoms and 214 individuals without ED symptoms were recruited and compared on levels of IU, insecure (anxious and avoidant) attachment styles, extraversion and openness. Secondly, the contribution of these factors to IU were tested.

**Results:**

Compared to the non-ED group, the ED group scored higher on IU, insecure attachment, and lower on extraversion and openness. Regression analyses confirmed that higher insecure attachment, and lower extraversion predicted higher IU scores in the ED group, and that insecure attachment predicted higher IU scores in the non-ED group.

**Conclusions:**

Results confirm the relevance of IU to ED, and demonstrate that personality traits and insecure attachment styles contribute to IU in ED. Findings add to the growing literature on IU in ED and suggest that people with EDs may benefit from clinical interventions targeting IU.

## Plain English summary

Studies show that people with eating disorders (ED) do not tolerate uncertainty about the future well and that uncertainty makes them feel anxious. To understand whether personality traits and attachment styles contribute to this intolerance of uncertainty (IU) in people with EDs, our study included a group of people with ED symptoms and a group without ED symptoms. We found that the ED group had higher levels of IU than people in the non-ED group. Also, we found that the ED group reported more insecure attachment styles and less extraversion and openness personality traits. Lastly, we found that in the ED-group, higher insecure attachments and lower extraversion contributed to higher IU, and that insecure attachment contributed to higher IU in the non-ED group. This study suggests that people with EDs may benefit from learning to tolerate uncertainty and that future research should examine possible treatments targeting IU for people with ED and ED symptoms.

## Background

Eating disorders (EDs), including anorexia nervosa (AN), bulimia nervosa (BN) and binge eating disorder (BED), have the highest mortality rates of all mental health disorders [1, 2], treatment interventions are suboptimal, relapse rates are high and 20% of patients become chronically ill [3–5]. Given the complexity of these potentially life-threatening disorders, a multi-faceted and interdisciplinary approach to understanding EDs is required. Indeed, the integration of genetic factors with biological and social-psychological models is needed to enhance current knowledge [1]. Not only are comorbid symptomatologies (e.g. anxiety, depression) inherent to EDs [6], most individuals with EDs experience difficulties across various life domains (i.e. work/school, interpersonal relationships and general cognitive functioning such as emotion processing [7–9]. This indicates that alongside identifying ED-specific symptom manifestations like weight, diet and exercise [10], it is important to study more general psychological vulnerabilities and transdiagnostic processes contributing to the development and maintenance of EDs.

A growing body of theoretical and empirical research suggests that individuals with EDs display and report psychological inflexibility (for a theoretical review see Merwin and colleagues [11]). Cognitive rigidity, a need for control and structure [12, 13] and high levels of perfectionism [14, 15] found in individuals with EDs are associated with more severe symptoms and are thought to hinder treatment [16]. Intolerance of uncertainty (IU) is a cognitive factor suggested to contribute to this inflexibility.

### Intolerance of uncertainty

IU is defined as “a dispositional characteristic that results from a set of negative beliefs about uncertainty and its implications and involves the tendency to react negatively on an emotional, cognitive, and behavioural level to uncertain situations and events” (p.216) [17]. Originally studied as a predictor of worry and general anxiety disorder (GAD [18]), in the last decade researchers have recognised IU as a predictor for a wide range of psychopathologies, including anxiety and depression [19–21], obsessive-compulsive symptoms [22], emotional problems [23, 24] and psychosis [25]. The literature on IU and its associated psychopathologies is fast growing, providing evidence for cognitive, behavioural and neural correlates of IU [26]. For example, those with high IU display attentional biases towards uncertain/ambiguous stimuli [27], display abnormal decision making processes (such as an increase in requirement for evidence before making decisions) [28], and perform poorer on basic behavioural tasks such as typing [29]. Moreover researchers have identified possible neural correlates (e.g., the amygdala, vmPFC, DLPFC,  anterior cingulate cortex, and orbitofrontal cortex) [30]. Furthermore, referring to IU as a “fear for the unknown”, recent theoretical papers [31] place IU at the core of psychopathology, in particular for those individuals with anxiety-related disorders such as EDs [32].

### Intolerance of uncertainty and EDs

Given the transdiagnostic nature of IU and the high rates of comorbid psychological disorders such as those associated with IU, it is unsurprising that ED researchers have started investigating IU in EDs. A growing body of studies support the relevance of IU for people with EDs [13, 33–38]. The importance of IU for AN was established initially in a qualitative study, in which AN patients reported experiencing uncertainty as extremely stressful and described trying to avoid uncertainty where possible [13]. Empirical studies have since identified clinical levels of IU in AN and BN [34, 35], comparable to those with anxiety disorders [34]. All published studies confirm associations between higher levels of IU and more severe (self-reported) ED pathology. Moreover, there is initial evidence that IU is associated with anxiety and depression in EDs [36–38] as well as with other comorbid emotional difficulties such alexithymia [37].

### Predicting intolerance of uncertainty

Whilst evidence for IU as a vulnerability factor for EDs is building, there is little understanding as to which factors might lead to high IU in the context of EDs. It is important to understand these factors because they could be useful treatment targets. It has been hypothesized more broadly that attachment difficulties may fuel a need for certainty and control, and an intolerance for uncertainty, in that insecure attachment styles can cause individuals to perceive the world as a dangerous place, and insecurely attached individuals may not have adequate resources to cope with uncertain events [39]. A central tenet of attachment theory is the psychological construct of a secure base [39, 40] from which a child can explore the world confidently. It follows that people who develop an insecure attachment style are likely to be less inclined to explore uncertain situations. Insecure adult attachment is best understood as comprising continuous dimensions, with two intersecting axes [41]. On one axis lies anxious attachment i.e. undue sensitivity to the threat of relationship loss, resulting in a dependency. Anxiously attached adults are characterised by an inchoate and incoherent sense of self. On the other axis lies avoidant attachment and adults with this attachment style typically need their independence and self-sufficiency. They avoid emotional interactions and have impoverished internal experiences [42]. There is some evidence to suggest that anxious and avoidant attachments relate to different symptom expressions and mechanisms underlying these symptoms [43]. For example, one study found that the association between avoidant attachment and symptom reporting was mediated by emotion-focused coping [44] whilst another study found that the relationship between anxious attachment and symptom reporting was due largely to negative affectivity [45].

Attachment insecurity occurs in about 42% of the general population but a recent study estimated the comorbidity with EDs to be as high as 96–100% [46]. A small number of reviews on attachment and EDs have been published highlighting the common presence of insecure attachment styles in populations of people with EDs [47–50]. Consistent with attachment theory, Tasca and colleagues [51] showed that attachment insecurity (i.e. attachment anxiety and attachment avoidance) may lead to negative affect and in turn to body dissatisfaction and restrained eating in women with EDs. Another study [52] confirmed that insecure attachment was associated with body esteem, whereby a direct effect for attachment anxiety, and an indirect effect (via alexithymia) for avoidance attachment were found. Also, in young people, insecure attachment styles have been associated with ED symptoms [53]. To date, no study has explored whether an insecure attachment is associated with IU in individuals vulnerable to ED symptoms.

In addition to attachment styles, it has been hypothesised that personality traits may contribute to IU [54]. In particular, higher levels of IU seem to be associated with lower levels of openness to experience and extraversion [54, 55]. Openness to experience refers to levels of curiosity and independent judgment defined by being adventurous, imaginative and free-spirited. Low scores might be expected in IU, indicating a constricted range of interests. Extraversion refers to degrees of sociability and positive emotionality defined as gregariousness and assertiveness [56]. Low scores might be expected in IU, indicating introversion and social isolation. Berenbaum and colleagues [54] found a significant association between low openness scores and higher IU, and the authors speculated that closed mindedness might help account for why individuals avoid unpredictable circumstances.

Personality pathologies are very common in people with EDs [57]. Indeed, it appears likely that personality traits are implicated in the aetiology of EDs [58, 59], with one recent study demonstrating that personality explains up to 25% of the variance in EDs and general psychopathology in non-anorexic ED patients [60]. Moreover, personality traits can predict variations in treatment outcome in AN and BN patients [61–63]. Specifically, low levels of extraversion seem to contribute to distress associated with having an ED [64], and have been identified as a partial risk factor for AN, BN and BED [65]. Tasca and colleagues [64] found a constricted range of interests (i.e. low openness) explained a moderate amount of variance in ED attitudes and behaviours in women with an ED. However, to date, no study has explored whether personality factors might help to explain high levels of IU in people vulnerable to ED symptoms.

#### Aims

In sum, whilst IU is relevant to gaining a better understanding of ED symptoms, no study has yet examined the factors contributing to IU in people vulnerable to engaging in ED behaviours. Therefore, the aim of the current study was to investigate whether personality factors and attachment styles may explain a degree of the variance in IU in a population of women exhibiting ED symptoms.

The first hypothesis was that compared to women without ED pathology (non-ED group), women with ED pathology (ED group) would have higher levels of IU and anxious and avoidant attachment styles and lower levels of extraversion and openness.

The second hypothesis was that within the ED group, higher levels of anxious and avoidant attachment styles, and lower levels extraversion and openness would be significant predictors of IU. To explore the specificity of this finding, this model will also be tested in the non-ED group.

## Methods

### Participants

The target population was women aged 16 or over able to read and respond in English. Convenience sampling and snowballing strategies including face-to-face sampling within Regent’s University London, and online via personal, social and professional networks (e.g. Facebook, LinkedIn) were used to recruit participants. Purposive sampling was also used in which places where people affected by EDs were likely to be present were targeted, including an ED conference in London, through advertising on the website and mailing lists of the ED  charity Beat UK, and via online ED groups worldwide (e.g. Google + communities, Facebook). Original data were collected from 809 people and all surveys not fully completed were discarded (*n* = 237, age range = 1–71 years). Two participants were excluded due to not  meeting the inclusion criteria for age and seven were excluded due to not meeting the inclusion criteria for gender. Fourteen participants met the criteria for outliers on the age variable and were excluded. This resulted in a final sample of 549 women (age range = 16–71 years, *M* = 34.92, *SD* 12.5) (see Table 1).

**Table 1:** Descriptive statistics for the Eating Disorder and Non-Eating Disorder Symptom Groups

	non-ED group (n = 451)				ED group (n = 98)				Test Statistics		Confidence Intervals
	Min	Max	M	SD	Min	Max	M	SD	*p*	*Cohen’s D*	
Age	16.00	74.00	35.53	12.57	16.00	55.00	32.15	11.58	< .02	0.28	−2.70, −2.38
EDE-Q	.00	3.87	1.75	1.12	3.90	5.90	4.81	.59	< .001	2.63	.92, 5.46
IUS	27.00	128.00	60.78	22.73	329.00	135.00	96.84	26.81	< .001	1.50	−41.14, −30.97
Anxious Attachment	6.00	42.00	22.52	7.59	10.00	42.00	27.19	7.86	< .001	0.60	−6.34, −3.00
Avoidant Attachment	6.00	39.00	17.71	7.35	6.00	42.00	23.57	8.75	< .001	0.73	−7.53, −4.20
Extraversion	1.25	4.83	3.32	0.61	1.25	4.58	2.829	0.71	< .001	0.69	.02, .61
Openness	2.27	5.00	3.77	0.53	1.91	4.55	3.50	0.57	< .001	0.49	.03, 53

*Abbreviations*: *ED* Eating Disorders, *M* Mean, *SD* Standard Deviation, *EDE-Q* Eating Disorder Examination, *IUS* Intolerance of Uncertainty Scale

Using data from the Eating Disorder Examination Questionnaire (EDE-Q [66]), two groups were created using a cut point of 1SD above the mean (3.88). One group, referred to as the ED group (EDE-Q score > 3.88) comprised of 98 individuals and the other group, referred to as the non-ED group (EDE-Q score < 3.88) comprised of451 individuals.

### Measures

#### Eating disorder examination questionnaire (EDE-Q 6.0)

The EDE-Q 6.0 [67] is a 28-item measureused as a screening tool for ED psychopathology and has excellent discriminant validity [68]. It retrospectively assesses the frequency of thoughts, feelings and behaviours over the previous 28 days. Participants are asked to respond on a 7-point Likert scale ranging from 0 to 6. The outcome variable used was the EDE-Q global subscale [68], which is created by summing and averaging other subscales to produce a score between 0 and 6 and provides an overall estimate of self-reported ED symptoms. Higher scores indicate a greater level of pathology and the global subscale is used in both clinical and community studies [69]. The Cronbach’s α value in the present sample was .96, implying excellent internal consistency [70].

### Intolerance of uncertainty scale (IUS-27)

The IUS-27 [71, 72] has 27 items that assess cognitive-behavioural and emotional reactions to ambiguous situations and the consequences of uncertainty, as well as attempts to control future events. Items are in the form of propositional statements e.g. “When it’s time to act, uncertainty paralyses me” (a cognitive-behavioural item), and “Unforeseen events upset me greatly” (an emotional item). Item responses are based on a 5-point Likert scale from 1 (*Not at all characteristic of me*) to 5 (*Entirely characteristic of me*). Items are summed to produce a total, with scores ranging from 27 to 135. Higher scores are indicative of higher levels of IU. IUS-27 has been demonstrated to have reliability and validity in different cultures [73] so was suitable for this study’s international sample. The Cronbach’s α value in the present sample was .97, implying excellent internal consistency [70].

### Experience in close relationships-revised scale short form (ECR-S)

The ECR-S [74] has 12 items in the form of statements designed to assess individual differences with respect to insecure attachment. Insecure attachment can be split into two further factors: anxious attachment and avoidant attachment [75]. Anxious attachment encompasses fear of rejection and the need for approval, whilst avoidant attachment reflects fear of intimacy and dependence. Items include “My desire to be very close sometimes scares people away” (i.e. anxious) and “I try to avoid getting too close to my partner” (i.e. avoidant). Item responses are on a 7-point Likert scale from 1 (*Strongly disagree*) to 7 (*Strongly agree*). Scores range from 12 to 42 per factor and higher scores are indicative of greater insecurity. For the six items of the anxious scale and for the six items of the avoidant scale the Cronbach’s α values in the present sample were respectively .78 and .83,  implying acceptable to good internal consistency [70].

### NEO personality inventory (NEO-FFI)

The personality factors ‘Extraversion’ (E) and ‘Openness to Experience’ (O) were assessed using the E and O scales, of the NEO Five-Factor Inventory (NEO-FFI, Adult Version [56]). Extraversion assesses a person’s levels of activity, gregariousness and positivity, whereas Openness assesses aspects of curiosity and conservativeness. Higher scores on the 5-point Likert scale ranging from 1 (*Strongly disagree*) to 5 (*Strongly agree*) indicate higher levels of extraversion or openness to experience. In the present sample the α value for E was .83 (12 items) implying good internal consistency, and for O was.70 (11 items), implying acceptable internal consistency [70].

### Procedure

Ethical approval was obtained from Regent’s University London (Ref 15.28) and the survey was made available at https://nl.surveymonkey.com/ for 11½ weeks from 1st May 2015. Participants were first presented with information about the study and asked to provide informed consent if they wished to proceed. They were then asked to provide demographic details (age and gender). After this, they completed the EDE-Q 6.0, IUS-27, NEO-FFI and ECR (S). Participation lasted approximately 20 min, with a full written debrief provided on completion. The research was conducted in keeping with the Declaration of Helsinki (2013).

### Data analysis

After confirming the data were normally distributed, t-tests were used to assess group differences for all measures. Subsequently, correlation analyses were run for the ED group and non-ED group separately. Lastly, linear regression analyses were conducted for the ED group and non-ED group with IUS scores as the dependent variable, and ECR anxious attachment scores, ECR avoidant attachment scores and NEO Extraversion scores as predictors for the ED group; and  ECR anxious attachment scores, ECR avoidant attachment scores and NEO Extraversion scores and NEO openness scores as predictors for the non-ED group. Effect size estimations were calculated using Cohen’s *D*, with 0.2 indicating a small effect, 0.5 a medium effect and 0.8 a large effect [76].

## Results

The non-ED group was significantly older than the ED group (*t* = 2.443, *p* < .02, *D =* 0.28).The ED group had significantly higher levels of self-reported ED pathology, measured using the EDE-Q (*t* = −26.218, *p* < .001, *D =* 2.63) and IU, measured using the IUS compared to the non-ED group (*t* = −13.933,*p* < .001, *D* = 1.50). As expected, given the literature reviewed earlier, significant differences emerged on all other predictor variables, with small to large effect sizes (see Table 1).

### Correlations in the ED group

There was a significant positive correlation between IU score and the ECR anxious and ECR avoidant subscales (effect sizes medium to large), whereby more anxious and avoidant attachment styles were associated with higher levels of IU (see Table 2).

**Table 2:** Correlations in ED group

	*Age*	*EDE-Q*	*IUS*	*Anxious attachment*	*Avoidant attachment*	*Extraversion*	*Openness*
*Age*	*-*		*-*				
*EDE-Q*	*r = −.315,* *p < .002*	*-*					
*IUS*	*r = −.066,* *p = .520*	*r = .374,* *p < .001*	*-*				
*Anxious attachment*	*r = −.186,* *p = .067*	*r = 262,* *p < .001*	*r = .381,* *p < .001*	*-*			
*Avoidant attachment*	*r = −.065,* *p = .527*	*r = .182,* *p = .073*	*r = .340,* *p < .001*	*r = .112,* *p = .271*	*-*		
*Extraversion*	*r = −.030,* *p = .772*	*r = −.208,* *p = .040*	*r = −.469,* *p < .001*	*r = .012,* *p = .907*	*r = −.294,* *p < .003*	*-*	
*Openness*	*r = −.059,* *p = .565*	*r = .041,* *p = .688*	*r = −.096,* *p = .348*	*r = −.028,* *p = .787*	*r = −.223,* *p < .03*	*r = .217,* *p < .04*	*-*

There was a significant negative correlation between IU scores and the NEO extraversion and NEO openness subscales (effect sizes medium to large), whereby lower extraversion and lower openness were associated with higher levels of IU (see Table 2).

### Predicting IU in ED group

A multiple regression analysis was run with ECR anxious and ECR avoidant subscales and NEO extraversion and NEO openness subscales as predictor variables and IUS scores as dependent variable, controlling for age including the ED group only. The model accounted for 39.7% of the variance in IU scores (R^2^ = .397, F(3,94) = 20.638, *p* < .001).

Both anxious and avoidant attachment significantly predicted IU scores (anxious: b = .367, t(451) = 4.546, *p* < .001, avoidant: b = .175, t(451) = 2.070, *p* < .041). Extraversion also significantly predicted IU scores (b = −.422, t(451) = −5.029, *p* < .001).

To explore the specificity of these findings, the same analyses were conducted within the non-ED group.

### Correlations in non-ED group

There was a significant positive association between IU scores and the ECR anxious and ECR avoidant subscales (effect sizes medium to large), whereby more anxious and avoidant attachment styles were associated with higher levels of IU (see Table 3).

**Table 3:** Correlations in non-ED group

	*Age*	*EDE-Q*	*IUS*	*Anxious attachment*	*Avoidant attachment*	*Extraversion*	*Openness*
*Age*	*-*						
*EDE-Q*	*r = − .162,* *p < .001*	*-*					
*IUS*	*r = −.295,* *p < .001*	*r = .487,* *p < .001*	*-*				
*Anxious attachment*	*r = −.198,* *p < .001*	*r = .306,* *p < .001*	*r = .500,* *p < .001*	*-*			
*Avoidant attachment*	*r = −.038,* *p = .424*	*r = .141,* *p < .01*	*r = .205,* *p < .001*	*r = .173,* *p < .001*	*-*		
*Extraversion*	*r = .084,* *p = .076*	*r = −.163,* *p < .001*	*r = −.433,* *p < .001*	*r = −.216,* *p < .001*	*r = −.201,* *p < .001*	*-*	
*Openness*	*r = .152,* *p < .001*	*r = −.082,* *p = .083*	*r = −.180,* *p < .001*	*r = −.059,* *p < .05*	*r = −.141,* *p = 209*	*r = .217,* *p < .001*	*-*

There was a significant negative association between IU scores and the NEO extraversion and NEO openness subscales (effect sizes medium to large), whereby lower extraversion and lower openness were associated with higher levels of IU (see Table 3).

### Predicting IU in the non-ED group

Again, a multiple regression analysis was run with ECR anxious and ECR avoidant subscales and NEO extraversion and NEO openness subscales as predictor variables, and IUS scores as dependent variable, controlling for age. The model accounted for 37.0% of the variance in IU scores (R^2^ = .370, F(4446) = 65.510, *p* < .001).

Anxious attachment significantly predicted IU scores (anxious: b = .420, t(451) = 10.831, *p* < .001). Extraversion and openness also significantly predicted IU scores (extraversion: b = .315, t(451) = −7.866, *p* < .001; openness: b = −.080, t(451) = −2.049, *p* = .041.

Avoidant attachment did not predict IU scores (b = .043, t(451) = 1.102, *p* = .271).

## Discussion

This study aimed to explore the relationship between IU, insecure attachment styles (namely anxious and avoidant) and personality factors (namely extraversion and openness to experience) in individuals with high levels of ED cognitions, assessed using online data collection. The first hypothesis was supported by the data, with medium to large effect sizes, i.e. that women reporting higher levels of ED pathology would have higher levels of IU, more anxious and avoidant attachment styles and lower levels of extraversion and openness to experience than women with lower levels of ED pathology. The second hypothesis was partially supported by the data, i.e. for those with EDs, higher levels of anxious and avoidant attachment styles and lower levels extraversion and openness would be significant predictors of IU, with the model explaining 46.6% of variance in IU. While anxious and avoidant attachment and extraversion significantly predicted IU scores, openness to experience did not. This finding was somewhat specific to the ED group, as the same model applied to the non-ED group predicted less of the variance in IU (31.2%). Moreover, unlike the ED group, avoidant attachment was not a significant predictor of IU scores for the non-ED group even though two of the predictors were also important in the non-ED group (i.e. anxious attachment and extraversion) and, similar to the ED group, one was not (i.e. openness to experience). These findings suggest that high levels of anxious attachment and low levels of extraversion may be factors that contribute to IU in the general population of females, but that an avoidant attachment may be a specific risk factor which contributes to IU in those individuals with high levels of ED cognitions.

In conclusion, these findings suggest that attachment and personality traits may contribute to the high levels of IU that have been identified in individuals with high levels of ED cognitions in previous studies of individuals with EDs [33–38]. This raises the question as to whether IU may function as a moderating or mediating factor in the relationship between attachment styles, personality traits, ED-related symptoms and general functioning in those with an ED. This is an important question to answer, as it may change our focus of targets in ED treatment, and should be followed up in future research using clinical populations.

### Clinical implications

Recovering from EDs requires facing uncertainty, trying new things and building new relationships. These findings suggest that individuals with ED cognitions may find these tasks more difficult than the non-ED population and it could be suggested that before engaging in intensive, evidence based treatments such as family therapy [77] and CBT [15], patients may benefit from a treatment adjunct which helps them to tolerate uncertainty, possibly through building supportive relationships and developing greater confidence to connect and spend time with others, given the findings of the regression model. Another possibility may be to support patients by adding a module targeting IU to established treatments, an approach taken by the Maudsley Model of Anorexia Nervosa Treatment for Adults (MANTRA) alongside other modules include training flexibility and social-emotional skills [16].

Indeed, alongside the growth in theoretical work related to IU, there have also been advances in  the development of IU-focused clinical interventions. A number of randomized clinical trials have tested a cognitive-behavioural intervention as developed by Dugas and colleagues and found moderate to large effects [78–80]. Moreover, there is evidence that reductions in IU may drive reductions in disorder-specific and transdiagnostic symptoms (e.g. anxiety and depression) across patient groups [24, 81]. To the authors’ knowledge, only one study has examined targeting IU in an ED population, and found that targeting IU in adolescent AN inpatients resulted in a reduction in IU and the treatment was experienced as being useful and acceptable by patients (Sternheim LC, Harrison A: The Acceptability, Feasibility and Possible Benefits of a Group-Based Intervention Targeting Intolerance of Uncertainty in Adolescent Inpatients with Anorexia Nervosa, submitted). More research is necessary to corroborate these findings. To conclude, it is important to continue increasing our understanding of the role of IU for individuals with high levels of ED cognitions, to pave the way to new treatment possibilities.

### Limitations

Although this study involved a control group to explore the specificity of its findings and reports on the largest sample to date in which associations between IU and EDs has been investigated, there are a number of limitations to note which could be addressed in future work. The participants in the sample self-reported their symptoms of EDs and the study did not attempt to conduct a psychiatric assessment to confirm a diagnosis and therefore any conclusions drawn may not be translatable to clinical populations. Indeed, not asking participants whether they have ever been diagnosed with, or sought treatment for, an eating disorder or another mental health condition may further limit possible generalisation to clinical populations. However, it is of note that the group of individuals with significant ED cognitions in this sample reported a mean score (mean = 3.26, SD = 1.17) similar to individuals of a similar age (mean = 2.97, SD = 0.65) identified as cases in Mond et al.’s community sample validation study of the EDE-Q [82], suggesting that the ED group in this analogue study may be representative of those with clinically recognized EDs. This suggestion emphasises the significance of the findings for individuals with clinically significant ED cognitions and warrants future work to replicate these findings in confirmed clinical populations. This study adopted a transdiagnostic perspective and did not differentiate between specific diagnostic subtypes (e.g. AN and BN) which may have been interesting in terms of exploring the degree to which the relationships were associated with specific types of symptoms.

## Conclusion

This study provides further evidence for the importance of IU in people with EDs. Moreover, this study demonstrates that personality traits, in particular lower levels of extraversion, and anxious and avoidant attachment styles contribute to IU in people vulnerable to EDs. Findings suggest that people with EDs may benefit from clinical interventions targeting IU, potentially before or alongside existing evidence-based treatments for ED. Future research should aim to confirm the identified associations in clinical samples, and explore the effectiveness of IU-based interventions in EDs.

## Abbreviations

AN: Anorexia nervosa
BN: Bulimia nervosa
CBT: Cognitive behavioral therapy
EDs: Eating disorders
EDE-Q: Eating disorders examination questionnaire
ERC: Experience in close relationships
GAD: Generalized anxiety disorder
IU: Intolerance of uncertainty
IUS: Intolerance of uncertainty scale
OCD: Obsessive compulsive disorder
